# Diagnostic and vaccination challenges in the PPR pre-eradication era: Identifying gaps and potential unintended consequences

**DOI:** 10.5455/javar.2025.l987

**Published:** 2025-12-25

**Authors:** Reham Karam, Yahya Aljasem, Saleh Alrashedi, Ali Alhafufi, Mohammed Abuhaimed, Hassan Albaqshi

**Affiliations:** 1 Department of Virology, Faculty of Veterinary Medicine, Mansoura University, Mansoura City, Egypt; 2 Virology department, Weqaa Central Laboratory, Weqaa Centre, Riyadh, Saudi Arabia; 3 Weqaa Centre, Riyadh, Saudi Arabia; 4 Weqaa Central Laboratory, Weqaa Centre, Riyadh, Saudi Arabia

**Keywords:** Peste des petits ruminants virus, PPR eradication, PPR mass vaccination, unintended consequences of eradication

## Abstract

Peste des petits ruminants (PPRs) is a transboundary, highly contagious, notifiable, viral disease of small ruminants. In recognition of its global threat and socioeconomic impact, World Organisation for Animal Health (WOAH) and Food and Agriculture Organization (FAO) have targeted PPR for eradication by 2030, accelerating the need for robust diagnostic, surveillance, and vaccination strategies. This review synthesizes global efforts toward PPR eradication, with a particular focus on diagnostic efficacy, vaccination coverage, and the persistent challenges that hinder progress. It also addresses concerns raised in the 2024 WOAH/FAO technical review regarding potential unintended consequences of eradication—namely, the ecological niche left by rinderpest and PPR that may allow the emergence or spread of other morbilliviruses. A literature search was conducted using peer-reviewed articles (2015–2025) and recent FAO/WOAH reports. Key gaps were identified in vaccine deployment, Differentiating Infected from Vaccinated Animals capability, and field diagnostics in addition to the potential unintended consequences of eradication. Finally, we advocate for the integration of predictive modeling to assess the risk of disease reintroduction and host spillover and for embedding these insights into future eradication policies.

## Introduction

Peste des petits ruminants (PPRs) is a lymphotropic and epitheliotropic disease of major veterinary and economic significance. PPR causes high morbidity (up to 100%) and mortality (up to 90%). The direct economic losses from PPR epizootics are estimated to reach USD 2 billion annually. In addition to these losses, large-scale vaccination campaigns impose a significant financial burden. Recent estimates indicate that approximately 1.7 billion susceptible small ruminants—representing up to 80% of the global population—remain at risk of PPR [1].

It is caused by the Peste des petits ruminants virus (PPRV), which belongs to the genus *Morbillivirus*, within the family *Paramyxoviridae* and the order *Mononegavirales*. The virus was previously referred to as small ruminant morbillivirus, reflecting its close relationship with other morbilliviruses, such as rinderpest virus, canine distemper virus, feline morbillivirus, and measles virus [2,3].

PPR affects sheep and goats, where goats are more severely affected. Other species can also be affected, such as cattle, buffalo, camels, and pigs [4]. It is thought that cattle and camels are dead-end hosts, as they can contract infection and seroconvert without being able to spread the virus or excrete it [5]. A study used an *in silico* approach to gain insight into the potential host range of PPR. It compares the signaling lymphocytic activation molecule (SLAM) receptor sequence that is used by PPRV to enter the host cell. Surprisingly, nine families, including Bovidae, Camelidae, Elephantidae, Suidae, Cervidae, Felidae, Muridae, Canidae, and Ceratopogonidae, have been found to be susceptible to PPRV infection but require further experimental characterization [6]. Wild ruminants can serve as maintenance hosts, bridge hosts, or dead-end hosts [7–12]. Despite being highly contagious, PPRV has limited potential for indirect transmission due to its relatively large particle size (approximately 400–500 nm) and intrinsic susceptibility to environmental degradation, which are typical characteristics of viruses within the *Paramyxoviridae* family. Transmission predominantly occurs through direct contact with infected animals within the herd via aerosolized respiratory secretions or the ingestion of contaminated material (alimentary route) [13,14].

During outbreaks in naïve herds, PPR often results in high morbidity and mortality rates, whereas lower mortality is generally observed in endemic areas. Recent studies have shown that different PPRV strains can result in varying clinical outcomes. For example, the Morocco strain has been shown to be highly virulent, whereas the Côte d’Ivoire 1989 (IC89) strain caused only mild infection in Saanen goats [15]. PPR is the next virus in the list for complete eradication by 2030, according to WOAH/FAO estimates.

This review aims to assess the current status of the PPR control strategy in terms of vaccination and diagnosis, with the goal of identifying weaknesses and potential post-eradication consequences.

### Ethical approval

This article does not contain any studies with human participants or animals performed by any of the authors. Therefore, ethical approval was not required.

## Materials and Methods

This review was conducted using a narrative review methodology. A comprehensive literature search was performed to identify relevant peer-reviewed articles and online reports focusing on PPR, with specific emphasis on eradication strategies, diagnostics, and vaccination. Additional literature related to the consequences of virus eradication (e.g., smallpox, rinderpest, poliovirus, and disease modeling) was also included to support the research question raised.

Searches were conducted in PubMed (https://www.ncbi.nlm.nih.gov/pubmed/) and Google Scholar (https://scholar.google.com/), and Official documents and technical reports were obtained from the WOAH (https://www.woah.org/en/disease/peste-des-petits-ruminants/) and FAO (https://www.fao.org/ppr/en/) websites between January and June 2025, using combinations of the following keywords:

“Peste des petits ruminants,” “PPR virus,” “PPR eradication,” “PPR vaccination,” “PPR diagnosis,” “virus eradication,” “rinderpest,” “smallpox virus eradication,” “poliovirus eradication consequences,” and “modeling and PPR virus.”

The inclusion criteria comprised (i) articles published between 2015 and 2025, (ii) written in English, and (iii) addressing aspects of PPR or relevant viral eradication efforts. Exclusion criteria include: Opinion pieces, non-English documents, duplicates, abstracts without full text, or studies irrelevant to PPR control/eradication.

A total of 101 articles were included in the final synthesis. Although this is a narrative review, we emphasized clarity and relevance of evidence in line with the Scale for the Quality Assessment of Narrative Review Articles (SANRA criteria) [16].

## Results and Discussion

### PPR virus pathogenesis and clinical manifestations

Infected animals serve as a persistent source of infection for susceptible animals in proximity, namely, an “episystem” on certain occasions [17]. The rate of viral spread is influenced by various environmental factors, as outlined by [18]. The incubation period typically ranges from 4 to 6 days but may last up to 14 days in certain cases [19,20]. Recognizing the epidemiological role of atypical hosts in PPR spread is essential for the success of global eradication efforts and for minimizing the potential impact of the virus on wildlife conservation .

The clinical signs of PPR range from acute to subclinical forms. Acute cases are characterized by high fever, mucopurulent oculonasal discharge, necrotic oral lesions, pneumonia, and gastroenteritis, with watery to bloody diarrhea in advanced stages. Respiratory involvement may include coughing, pleural rales, and abdominal breathing. In severe infections, mortality can occur within 1 week [13].

### PPR virus nature and virus-host interaction

#### PPRV nature

Peste des petits ruminants virus is spherical to pleomorphic in shape and encloses an ssRNA of negative polarity. It is approximately 15,948 nt in length. The genome of PPRV consists of a 3’ noncoding region known as the genome promoter, followed by six structural protein-coding genes: nucleocapsid (N), phosphoprotein (P), matrix (M), fusion (F), hemagglutinin (H), and large polymerase protein (L). The 5’ end contains a noncoding anti-genome promoter region. The viral RNA is encapsidated by the N protein. Additionally, the *P* gene gives rise to two nonstructural proteins, V and C, through an RNA editing mechanism [21]. *F* and *N* genes are commonly used for genetic and phylogenetic analyses. Based on partial N or partial F gene sequencing, the virus is classified into four distinct genetic groups (I–IV), with only one immunological serotype [22]. Phylogenomic analyses revealed a complex relationship among PPRV lineage II (LII) strains, consistent with extensive transboundary circulation across West Africa. In contrast, lineage IV (LIV) sequences exhibited clear regional separation, with West and Central African strains forming a sister clade to other LIV sequences, suggesting an African origin. It is suggested that the divergence of modern LII and LIV strains occurred between the 1960s and 1980s—a period critical for PPRV diversification and global spread. Phylogenetic comparisons of historical and contemporary isolates from lineages I–III reveal high genetic diversity in Africa until the late 20th century, followed by possible bottleneck events that shaped viral evolution. Molecular evolution analyses further demonstrate that LII and LIV strains have been subjected to distinct selection pressures, with differences in codon usage and adaptive selection observed across all viral genes [23].

**Figure 1.**
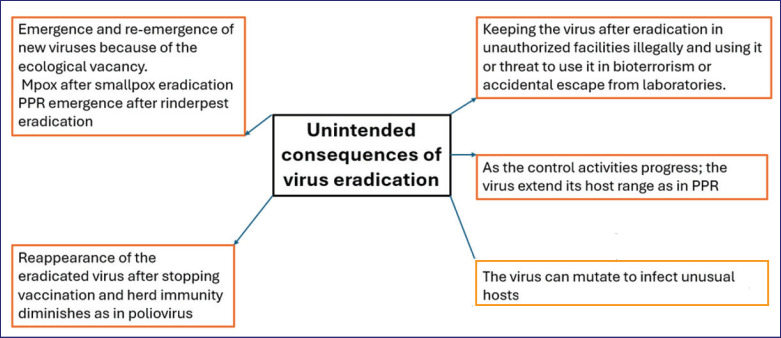
Morphology and genome organization of PPR virus showing it`s proteins.

In Figure 1 and Table 1, we summarize PPR viral proteins and their functions and relevance for vaccine production.

**Table 1.** PPRV proteins and their functions <a href=""><url>[21]</url></a>.

Viral protein	Protein function	Relevance as a diagnostic or vaccine target
Nucleocapsid Protein (N).	- Encapsidates viral RNA - Evades host innate immunity as it inhibits interferon production mediated by interaction with IRF3, thereby blocking its activation. PPR-coded N protein has a role in the induction of autophagy.	- Used as diagnostic antigen in PPR cELISA
Phosphoprotein (P, V/C)	- Viral nonstructural proteins. - It is Core component of RdRp complex by forming heterologous P–N–L tripartite complex. - Supports viral replication - It is essential in the cell cycle control. - Suppresses innate immune response by blocking IFN signaling.	Non relevant
Envelope matrix protein (M)	- Regulates virion assembly.	- Non relevant
Fusion protein (F)	- Involved in virus-host membrane fusion - Key to virus entry - Contributes to pathogenicity - Strongly immunogenic	- Major target for neutralizing antibodies - Included in recombinant and subunit vaccines
Hemagglutinin protein (H)	- Involved in assembly - Facilitates virus-host fusion - Promotes pathogenicity - Highly immunogenic - Enhances replication	- Key antigen in live attenuated vaccines - Major neutralizing epitope
Large polymerase protein (L)	- Core catalytic unit of RdRp - Responsible for RNA synthesis and posttranscriptional modification	

### PPRV-host interaction

Like other members of the *Morbillivirus* genus, PPRV exhibits both lymphotropic and epitheliotropic properties and is primarily transmitted through aerosolized droplets. As a result, efficient transmission typically requires close contact among animals within a herd. It is widely accepted that PPRV initially infects the epithelial cells of the respiratory tract. Then, the immune cells in the respiratory mucosa may capture viral particles from the airway lumen and transport them to T-cell-rich zones within local lymphoid tissues, where early viral replication is likely to occur [20]. PPRV replicates in epithelial or lymphoid cells that express nectin-4 and SLAM receptors, respectively. In the early stages of replication, the viral H protein binds to cellular receptors, activating conformational changes in the F protein to facilitate entry through receptor-mediated endocytosis, as reviewed in [21]. This review describes the process in detail, including the cellular machinery involved.

### PPR global distribution

Peste des petits ruminants (PPRs) was first identified in the early 1940s in Côte d’Ivoire (West Africa) and has since expanded across much of the globe. The disease is now endemic in large parts of sub-Saharan Africa, North Africa, the Middle East, and Asia, with the exception of Southeast Asia [20]. Several factors influence the transmission and prevalence of PPRV, including climatic and geographical conditions, as well as farming and husbandry practices. Europe has been considered free of PPR for decades. However, in 2024, outbreaks were reported in Greece and Romania—countries previously recognized as PPR-free. This re-emergence represents a serious threat to domestic sheep, goats, and susceptible wild ungulates, with morbidity rates reaching 100% and mortality rates as high as 80% in naïve populations [24]. In response, both countries have implemented control measures, including movement restrictions, zoning, and stamping out of infected herds. The source of these outbreaks remains unclear, raising concern that past eradication efforts could be undermined without proper epidemiological tracing. Given that PPR is still considered exotic in Europe, any outbreak in this region could result in significant direct losses due to the lack of herd immunity. Affected countries are likely to depend on vaccines developed and produced in Africa or Asia [25]. Additionally, earlier outbreaks in Georgia (2016) and Bulgaria (2018) underscored the ongoing threat from neighboring endemic regions [26]. Several epidemiological studies have focused on regional prevalence over defined timeframes, using statistical tools to identify hotspots and guide targeted interventions. These studies are critical for supporting global control and eradication strategies [13].

### Eradication plan for PPR

Following the eradication of rinderpest in 2011, PPRV emerged, prompting WOAH in 2014 to launch a global eradication program targeting 2030, which requires coordinated multisectoral collaboration [26].

The global PPR eradication program employs a four-stage, stepwise approach. Stage 1 assesses local epidemiology to establish baseline virus circulation. Stage 2 involves targeted mass vaccination to reduce the incidence and transmission of the disease. Stage 3 intensifies surveillance, strengthens veterinary services, and reinforces control to eliminate residual infection. In Stage 4, vaccination is suspended, and countries must provide robust evidence of virus absence at zonal or national levels before formally applying to the World Organisation for Animal Health (WOAH) for PPR-free status [27].

The eradication stages include achieving 5 technical elements per stage. These elements include diagnostics, surveillance, prevention and control, legal frameworks, and stakeholders’ involvement. To help countries self-assess their situation, a tool known as the PPR monitoring and assessment tool (PMAT) was developed by the GF-TADS [28].

As with any disease, eradication is feasible when policy, scientific, and technical challenges are addressed. The following major challenges are described: understanding the small ruminant production system, empowering research to support eradication, adapting laboratory testing to the need for eradication as in Differentiating Infected from Vaccinated Animals (DIVA)-based testing, improving the epidemiological understanding of the virus, defining the role of wildlife and other species in PPR epidemiology and its associated risk factors, optimizing vaccine delivery and novel vaccines, especially DIVA, developing better control of animal movement, heightening serological monitoring, understanding socioeconomic impact, and garnering funding and the political will for the eradication progress.

The cornerstone phase of any eradication program requires an efficacious vaccine supported by reliable and sensitive diagnostic tools. Vaccination is considered the key strategy guided by epidemiological surveillance [20]. In endemic settings, vaccination is complemented by controlling animal movement and rigorous quarantine and surveillance measures [25].

In this review, we focus on two aspects [29] of PPR control and eradication: diagnostic activities and vaccination against PPR, which serves as the primary tool for control, especially in endemic areas, and highlights recent updates in both aspects.

### PPRV diagnosis

### Field diagnosis

PPR can be diagnosed clinically by observing symptoms such as pyrexia, oculo-nasal discharge, stomatitis, gastroenteritis, and diarrhea. In cases resulting in death, necropsy followed by pathological investigation proves highly informative, showing the virus caused pathognomonic lesions in the lungs and large intestine [30–32].  It mimics several other infections, such as foot-and-mouth disease (FMD), bluetongue, and orf [33], and therefore it must be differentiated from them using PCR during diagnosis. The onset and progression of clinical signs vary according to many individual variations within the host [34]. The occurrence of coinfections in PPR-affected cases is not uncommon, and this may lead to a misdiagnosis. A clinical scoring scheme is proposed that includes 4 stages. Stage 4 indicates the greatest severity, as the animal exhibits decreased alertness, dehydration, and a lack of movement. Stages 2 and 3 are less severe, showing the previously mentioned clinical signs, and stage 1 indicates the mildest form [35].

### Laboratory diagnosis

The laboratory diagnosis of PPR serves as a cornerstone for both confirming infection and characterizing the virus. After sample collection and their preparation, PPR can be isolated in the Vero cell line and produce a marked CPE that includes syncytia formation and eventually cell lysis. This method can be used for PPR detection and as a preliminary test before its specific detection by known antibodies in a serum neutralization test [36]. Fetal ovine heart cells can also be used for the proliferation of viruses. These cells are unique, immortal cells developed by the Weqaa Center Cell Culture unit in Riyadh, Saudi Arabia [37].

### Serological tests

Highly specific cELISA assays targeting antibodies against the hemagglutinin (H) and nucleoprotein (N) of the PPR virus have been developed at CIRAD and the Pirbright Institute and are used for serosurveillance studies, usually in endemic areas [36]. These assays play crucial roles in serological surveillance by enabling the detection of virus-specific antibodies in sheep and goat populations [38]. Active and passive surveillance can be integrated to achieve an overall view of the PPR status in several countries, like Sudan, Bangladesh, and Ethiopia [38–41]. Serosurveillance studies add significant value to PPR control when combined with risk factor identification on the Episystem platform [19,42-44]. A novel assay uses the baculovirus-expressed truncated NP (PPRV-rBNP)-based ELISA as an alternative to using the live virus for long-term use in endemic and non-endemic countries. The recombinant PPRV-rBNP demonstrated strong reactivity with PPRV anti-N monoclonal and polyclonal antibodies, confirming that the expressed protein retained its original conformation. To assess its diagnostic potential, crude PPRV-rBNP was evaluated in an avidin–biotin ELISA, either as a coating antigen or as a standard positive control, using a validated reference panel. The findings indicate that PPRV-rBNP is a viable alternative to the *Escherichia coli*-expressed recombinant PPRV-NPN and, importantly, its use circumvents the requirement for live PPRV antigen in ELISA-based diagnostics [45]. Another study produced nine novel nanobodies engineered from alpacas, which are single-domain antigen-binding fragments derived from heavy-chain-only antibodies of the camelid family. These nanobodies could specifically recognize fully inactivated PPRV by ELISA. They exhibited remarkable sensitivity and speed in detecting PPRV via in-house ELISA, highlighting their potential to advance diagnostic approaches and offering a promising avenue for future therapeutic interventions against PPR [29].

Antigen detection ELISAs are also available in commercial and research settings [46]. The ability of PPR to infect a wide range of hosts, including Bovidae, Suidae, and Camelidae, complicates serodiagnosis and necessitates kit validation with a broad panel of sera from diverse hosts. The cross-reactivity potential between members of *G*. *Morbillivirus* can be used to produce novel kits [47].

The development of DIVA ELISA kits, which are commercially available for low-income countries with a DIVA vaccine, is mandatory to achieve successful eradication. While ELISA is practical, scalable, and well-suited for large-scale epidemiological studies, the WOAH has designated the virus neutralization test as the gold standard for international trade despite its complexity and difficulty in large populations [8,45].

### Molecular detection of PPR virus

It is well established with SOPs and protocols available at the CIRAD laboratory website and the WOAH manual. RT-PCR assays that are gel-based or fluorescence-based are available [12,36,48]. It amplifies the *N*, *F*, and *H* genes either fully or partially [27,49]. Lineage discrimination is usually done using partial Sanger sequencing after partial amplification of the *N* gene using the primer pairs NP3/NP4 that amplify 351 bp [50]. A recently developed qRT-PCR assay targeting the *H* gene enables specific detection of PPRV lineage IV with remarkable sensitivity, reliably identifying as few as six RNA copies. This tool addresses a critical need for rapid and accurate lineage IV diagnosis, particularly given its increasing burden across Europe, the Middle East, and Africa [51].

Full-genome sequencing is also well established for PPR virus through long- and short-read-based platforms [52-54]. Some sequencing protocols use initial PCR enrichment before sequencing using overlapping or gene-specific primers. This protocol is sometimes hindered by the fact that the region of the *M*-*H* genes is G-C rich and their amplification is difficult. The use of long-read primers and the addition of DMSO to the sequencing reaction yielded successful amplification [55]. A successful protocol for PPR nearly full-genome sequencing utilizes 8 pairs of overlapping primers that span the entire genome. This requires the implementation of 8 PCR runs before Illumina MiSeq sequencing can be done [56]. The CIRAD reference laboratory in France uses the Illumina dsCDNA synthesis protocol that can be done directly on tissue samples after depletion of the host genome using DNAse treatment [57]. The PPR research has benefited from advanced sequencing technologies, such as RNA-seq and next-generation sequencing, in understanding PPRV host interaction at a molecular level [58-61], DNA microarrays [62], and non-coding RNA profiling [61,63].

Even after decades of research on PPR diagnosis optimization, several key points still require attention. A WOAH scientific and technical review [17] identified three main diagnostic gaps in PPR, namely, the development and validation of serological and noninvasive methods adapted to atypical hosts (e.g., wildlife), the integration of field diagnostic tests in surveillance activities, and the confirmation of the efficacy and safety of DIVA vaccines with validated differential diagnostic tests.

### Types of vaccines against PPR

Live attenuated vaccines are available for PPR control. Although it can induce immune protection, an eradication program would greatly aid in the development of vaccines that allow for the differentiation of infected animals from vaccinated ones (DIVA vaccines) [26].

Although current live attenuated PPR vaccines confer long-lasting immunity, the initial induction of virus-neutralizing antibodies is too slow to prevent transmission among in-contact animals. The precise cellular targets that support replication of the attenuated virus remain incompletely characterized. However, intranasal (i/n) administration, which mimics the natural route of infection, is proposed to induce a faster and more robust immune response, as observed with other respiratory pathogens. Additionally, intranasal delivery presents significant practical advantages in field settings: it is noninvasive, environmentally friendly, and highly amenable to mass vaccination campaigns. Recent studies have shown strong local cellular immune responses in the respiratory tract following intranasal immunization with PPRV vaccine strains [20].

### Live attenuated vaccines

Live-attenuated PPRV strains, developed through serial passages in tissue culture from different circulating lineages, have long served as vaccines throughout Africa, the Middle East, and much of Asia. The initial successful adaptation of PPRV to cell culture was accomplished by Gilbert and Monnier, who used sheep embryo kidney epithelial cells for serial passaging [26]. Six different live-attenuated homologous vaccine strains called Nigeria 75/1 (lineage II), Sungri 96 (lineage IV), Arasur 87 (lineage IV), Coimbatore 97 (lineage IV), Titu (lineage IV), and 45G37/35-K PPR (lineage IV) are currently available [25] and are given subcutaneously in sheep and goats in endemic countries with great success [20]. The Nigerian PPR virus vaccine (75/1), developed in 1989, is a lineage II strain that is the most widely used to protect against PPR of all genetic lineages [64]. ‘Sungri 96’ is another live attenuated vaccine developed from the lineage IV virus isolated in India in 1996 for 59 passages. The virus was propagated in marmoset lymphoblastoid cells. It elicits a strong immune response that lasts for 6 years [65]. This vaccine came in second place after the Nigerian 75/1 vaccine in popularity and use.

These vaccines induce long-lasting immunity, persisting for at least 3 years postvaccination, as observed with the Nigeria 75/1 (lineage II) and Sungri 96 (lineage IV) strains. [64] reported that the Nigerian 75/1 strain causes persistent immunity in once-immunized animals for up to 3 years. Importantly, they provide cross-protection between lineages and elicit robust innate, humoral, and cellular immune responses, with no evidence of recombination among vaccine or field strains [23]. The live-attenuated PPRV vaccine strain (Nigeria 75/1) is potent and safe for the vaccination of “Deutsche Edelziege,” or white German goats, as no transmission of the vaccine virus to “in-contact goats” was confirmed [25].

The innate immune response is initiated when pattern recognition receptors detect pathogen-associated molecular patterns of the PPR virus, forming the first line of antiviral defense [25].

For a long time, the generation of a humoral immune response by a vaccine candidate was considered sufficient. For some years, the generation of a cellular response to PPRV has been considered almost as essential as the humoral response. Live vaccine strains have been shown to elicit both humoral and cellular immune responses [25,26].

Another live attenuated vaccine, prepared from the Sungri 96 lineage IV, is currently used in the field against PPR in India and is administered via both intranasal and subcutaneous routes. Protective immunity against all four lineages of PPRs was successfully induced [27].

In addition, a study conducted in Africa, where partial sequencing of the master seeds of 10 different vaccine manufacturers was performed, revealed 100% homology. The genetic stability of the Nigerian 75/1 vaccine over 3 decades of usage is revealed [15]. Although live PPR vaccination has great potential, some drawbacks have been revealed.

### Drawbacks of live attenuated vaccines for PPR

The live attenuated PPR vaccine should be given in endemic areas where keeping it in a cold chain can be challenging. This fact has stimulated numerous research trials focusing on the development of TT, or thermotolerant vaccines. The minimum requirements for TT vaccines were established in a meeting of the PPR GEP (Global eradication program) consortium in 2017. They determined that the TT vaccine should tolerate 2°C–8°C for 2 years, 25°C for 10 days, and 5 days at 40°C after dilution [65].

These vaccines are not DIVA vaccines, so they do not differentiate vaccinated from infected animals, which makes the surveillance process after eradication success a difficult task. Trials are being performed for DIVA vaccine production and DIVA ELISA. Most of these vaccines are still in the trial phase or rely on microarrays that are difficult to implement in national PPR laboratories in endemic areas. Two recombinant live-attenuated PPR vaccines were developed at the Pirbright Institute in 2020 and have been proven to elicit a strong immune response and are safe [65].

It is not uncommon for attenuated viruses to revert to virulence, which could cause an outbreak. This potential was studied extensively by [66], who used deep sequencing to compare the full genomes of both wild-type and vaccine strains after different passages in Vero cells. The authors suggest that decreasing the passage number is better for obtaining fewer variable viruses and decreasing their potential to revert to virulence.

Following the widespread administration of live attenuated vaccines, the diagnostic and surveillance value of PPR-specific antibodies in serum samples has decreased. Additionally, a study by Eloiflin et al. [15] detected PPRV RNA in lacrimal secretions 6–11 days post-vaccination, which suggests that PCR detection may not be a reliable indicator of infection. Concluding that control and surveillance, especially in epizootic outbreaks, should rely on both real-time RT-PCR and antigen detection ELISA results, and that detection only does not mean active infection, and that DIVA is urgently needed or accompanied by PCR with antigen detection ELISA and/or histopathology, which can be difficult on some occasions [67].

Additionally, the effectiveness of these vaccines depends on cold-chain preservation, which requires resources that are potentially lacking in localities where the PPR is endemic.

Therefore, great efforts have been made to develop alternative live attenuated vaccines to address the need for DIVA and thermotolerance for PPRV. On the contrary, in nonendemic regions, inactivated vaccines offer protection against homologous strains. It offers a considerable level of immunity, holding no risk of reversion to virulence [26].

FAO/WOAH recommendations on proper PPR vaccination are reported. In conclusion, some important factors should be considered during PPR vaccination, including the Epi-unit approach, which focuses on vaccination, sampling, and surveillance based on groups of animals called “epi-units” that encompass groups of animals with the same geographic and epidemiological conditions [68]. On the other hand, the principal challenges with PPRV vaccines lie in their thermolability, even in lyophilized form, necessitating strict adherence to a cold chain for effective field delivery. Over the past decade, this issue has garnered considerable attention, prompting collaborations between researchers and manufacturers that have yielded notable advances in vaccine stabilization. Promising developments include the introduction of stabilized liquid formulations designed to simplify vaccine distribution to the point of use. Furthermore, AU-PANVAC has established quality control systems for thermostable PPR vaccines, which are expected to enhance both vaccine quality and accessibility in endemic regions. A critical limitation of current vaccines is the absence of a DIVA capability, meaning that vaccinated animals cannot be serologically distinguished from naturally infected ones. While such a tool was not required for rinderpest eradication, its availability would streamline the final stages of PPR eradication and strengthen post-eradication monitoring. Several promising DIVA vaccine candidates have been developed; however, further evaluation is necessary to confirm their safety, efficacy, and durability of protection. Strategies under investigation include genetic modifications of vaccine strains to generate unique antibody profiles, as well as the use of viral vectors to express the PPRV H glycoprotein. The latter approach leverages the fact that vaccinated animals produce anti-H but not anti-N antibodies, allowing for differentiation using existing ELISA platforms [17].

### Combined vaccines

Combined vaccines targeting both PPR and sheep pox have been developed and tested experimentally, with promising outcomes in several countries, including India, Cameroon, Morocco, Ethiopia, Egypt, and Russia. These bivalent vaccines have demonstrated immunogenicity and efficacy comparable to their respective monovalent vaccines. Although effective immune responses have been observed, the current literature lacks comprehensive data on the duration of postvaccination immunity in animals immunized with these combined formulations. Nevertheless, the vaccine strains used in the development of these combined vaccines are known to be highly immunogenic, and when administered individually as monovalent vaccines, are capable of inducing protective immunity that lasts for a minimum of 1 year following a single immunization [64]. The use of combined PPR and goatpox virus vaccines stimulated the immune system in PPR but adversely affected the outcome of the goatpox virus vaccine [14].

### Inactivated vaccines against PPR

In non-endemic regions, inactivated PPR vaccines are typically favored due to their enhanced safety profile, particularly in mitigating risks associated with live attenuated vaccines, such as potential reversion to virulence or unintended viral spread. The inactivated Morocco/2008 strain has shown promising results, offering a safe and effective alternative that elicits a robust humoral immune response in vaccinated animals. It belongs to lineage IV and was isolated in 2008 from an outbreak in Morocco [26]. Inoculation with this binary ethyleneimine-inactivated virus was safe in rats and goats and induced humoral responses [26,27]. Binary ethyleneimine is a common method used to inactivate viruses by modifying their nucleic acids while preserving the structure of the epitopes. This helps trigger an effective immune response [69].

The inactivated PPRV vaccine, which uses Mantonide-01 gel, Montanide oil ISA 206, and Carbomer as an adjuvant, has been found to induce the immunological response in sheep up to 28 days and potentially more. These formulations perform homologous protection and elicit immunity similar to that afforded by live attenuated vaccines [70]. After receiving two injections, both the rats and the goats exhibited 100% seroconversion to another inactivated PPR vaccine, which was prepared with delta inulin and TLR9 agonist oligonucleotides as adjuvants. Adversely, the Morocco/2008 PPRV inactivated vaccine adjuvinated with delta inulin failed to trigger an immune response comparable to that of live vaccines [65]. Certainly, more research is needed to formulate stable and more efficient vaccines to address the need in non-endemic countries.

### Recombinant vaccines

Recombinant vaccines for PPR offer a significant increase in the fight against this highly infectious and economically damaging animal disease. These vaccines feature both efficient and targeted delivery, as they use new genetic engineering methods. Vector-based vaccines utilize either viral or bacterial vectors, such as capripox viruses or adenoviruses, which are genetically engineered to express specific proteins of the PPR virus, including the H (hemagglutinin) and F (fusion) proteins. These vaccines mimic natural infection without causing disease [48].

Rojas et al. [26] reported that recombinant protein vaccines utilize *E. coli*, yeast, or baculovirus as expression systems to manufacture specific PPRV proteins, which are then used to activate the desired immune responses. Kumar et al. [47] noted that these vaccines are effective in eliciting an immune response, with the specific antigenic sites of the PPR virus being the primary focus.

### DNA vaccines

This technique involves the introduction of plasmid DNA, which encodes specific PPRV antigens, into the host organism. New vaccines are designed to enhance PPR diagnostics by leveraging technology that enables the DIVA. Chimeric vaccines combine genes from many pathogens in an effort to protect against a variety of illnesses. Recombinant vaccines that express PPRV proteins, for example, have been developed and have demonstrated protective efficacy against infections caused by both PPR and *Capripoxviruses* [71]. This strategy offers more protection, particularly in areas where both illnesses are prevalent. Among the many benefits of these recombinant vaccination approaches is increased safety*.* The dangers associated with live attenuated vaccinations are decreased with vaccines made from recombinant proteins. Additionally, they exhibit enhanced stability [47]. According to Rojas et al. [72], this makes them more suitable for use in distant and resource-constrained areas, as they are more resilient under various storage conditions. They also make targeted immunity possible. Ultimately, recombinant PPR vaccination represents a crucial step toward establishing safe and effective PPR control methods. Their various modes of action, along with creative genetic engineering, make them valuable tools in animal disease control.

The first-generation adenoviral vectors, characterized by deletions in the E1 and E3 regions of the adenoviral genome, are replication-defective—capable of infecting host cells but unable to replicate. Second-generation vectors include additional deletions or inactivation in the E2 and E4 regions, which encode proteins essential for viral replication in host cells. These modifications increase biosafety by reducing the risk of generating replication-competent adenoviruses through recombination. However, this increased safety often comes at the cost of reduced vector immunogenicity. The third-generation adenoviral vectors, also known as “gutless” or helper-dependent vectors, involve the complete removal of the adenoviral genome, retaining only the inverted terminal repeats and the packaging signal necessary for vector assembly [26].

The adoption of live attenuated vaccines in PPR eradication is efficient and has several positive aspects, particularly in the production process, and provides long-lasting immunity that can last 3–6 years for the 75/1 and Sungri vaccines.

### Vaccination trials, efficacy evaluation studies 2019-2025

Table 2 shows the recent PPR vaccine evaluation trials. Vaccine seromonitoring studies were also conducted to evaluate the efficacy of the PPR vaccine in terms of protection [78].

**Table 2.** Shows the recent PPR vaccine evualtion trials.

Objective	Study method	Vaccine studied	Outcomes	References
The effect of PPR vaccine strain serial passage on virus virulence and reversion to virulanence	Deep sequencing and sequence analysis to reveal the difference between the patent strain and attenuated one.	The Nigerian 75/1 live attenuated vaccine	The findings indicate that even a limited number of mutations may substantially influence PPRV pathogenicity. Although the risk of virulence reversion of the attenuated Nigeria 75/1 strain during serial cell culture passages appears low, restricting passage numbers during vaccine production remains advisable.	[66]
Evaluate the post vaccinal effect after vaccination with live attenuated vaccine and challenge with 2 different field strains. It studied the clinical signs, immune repsone and shedding of the virus.	It evaluated the virulence of two PPRV strains (CI89 and MA08) in Saanen goats. While MA08 induced classical severe clinical signs, CI89 caused only mild disease,	Live attenuated based on Nigeria 75/1 attenuated strain was used before challenge with 2 filed strains	Underscoring strain-dependent differences in pathogenicity within this model. The study also highlighted the influence of inoculation route on disease outcome and demonstrated that ocular swabs outperform blood samples for viral detection. Building on this robust challenge model, the study tested the efficacy of PPR-VAC^®^ (BVI, Botswana) against MA08 and confirmed its ability to block viral excretion and markedly attenuate clinical signs.	[73,75]
To produce a promising vaccine canididate for PPR control and eradication.	The VLPs were administered to mice, goats, and sheep with two booster doses following primary immunization. Both constructs elicited robust humoral responses, reflected by elevated IgG1/IgG2a ratios. In all species, high titers of virus-neutralizing antibodies (VNAs) and H- and F-specific antibodies were detected, with Tibet/30 VLPs consistently inducing higher antibody titers than Nigeria 75/1 VLPs.	Virus like particles	Mouse studies revealed that Tibet/30 VLPs elicited stronger interleukin-4 and interferon-γ responses compared to Nigeria 75/1 VLPs, indicating enhanced immunogenicity.	[74,76]
To evaluate Montanide -01 gel; Carbomer, and Montanide oil ISA 206 as an inactivating agents for PPR vaccine production.	The three adjuvents were used and evualted in terms of safety and potency in producing neutralization and total antibody in sheep immunized.	Inactivated vaccine	The three inactivating agents gave strong total and neutralizing immune response in sheep that last for 28 days and more.	[77]
To detect the safety and potency of a live-attenuated vaccine given to goats by detecting their immune response either cellular or humoral.	The safety of the given PPRV vaccine was estimated in terms of the absence of PPR clinical signs, negative pen-side test , minimal viral genome detection by RT-qPCR in vaccinated goats, and no evidence of horizontal transmission to in-contact animals. Moreover, the robust humoral and cellular immune responses observed confirm the strong immunogenic potency of this vaccine.	Live attenuated vaccine.	The live attenuated vaccines are sfe and potent and suits their ogjective to eradicate PPR.	[25]
To evaluate a multivalent vaccine ’’vectored in LSD’’ against PPR	Same as previous one.	Multivalent capripoxvirus-vectored vaccine candidate	The vaccine protected sheep from developing clinical PPR and markedly reduced viral shedding by real-time RT-PCR analysis of oral and nasal swabs. Following challenge infection, sheep exhibited a strong anamnestic response, characterized by the production of PPRV-neutralizing antibodies.	[48,78,79] they did the same protocol for evaluation.
To evaluate the ARRIAH vaccine to control PPR.	Evaluated the safety and protective efficacy of the ARRIAH live-attenuated PPRV vaccine (lineage II) in Saanen and Nubian goats, using a virulent lineage IV Mongolia/2021 isolate for challenge. For benchmarking, two commercial vaccines based on the Nigeria 75/1 strain were included for comparison.	Live attenuated vaccine strain “ARRIAH	It vslidated the use of ARRIAH vaccine for PPR control as it is not yet validated by the WOAH.	[80]

### PPR adequate vaccination challenges in low- and middle-income countries (LMICs)

PPR eradication can be significantly hindered by economic factors. Mass vaccination is crucial for achieving herd immunity in PPR-susceptible populations. Although the adequate supply of LMIC is a challenge. The thermal instability nature of current live-attenuated PPR vaccines requires their shipment in a cold chain, which incurs considerable costs and should be taken into account during the development of the overall eradication endgame [79,80]. Another challenge is the lack of political will that can hinder the proper vaccination process. This negative impact can stem from a lack of proper communication with other nations regarding coordination and guidance for PPR eradication by 2030, including the application of tools such as the PMAT. Poverty, lack of resources, and the high cost of vaccination campaigns are fundamental factors that need to be overcome for the benefit of the entire world. This economic vulnerability is further compounded by other costly diseases in small ruminants, such as Pregnancy Toxemia. The socioeconomic factors in PPR vaccination are comprehensively reviewed in [81].

### Unintended consequences of eradication campaigns and how to prevent them

**Figure 2.**
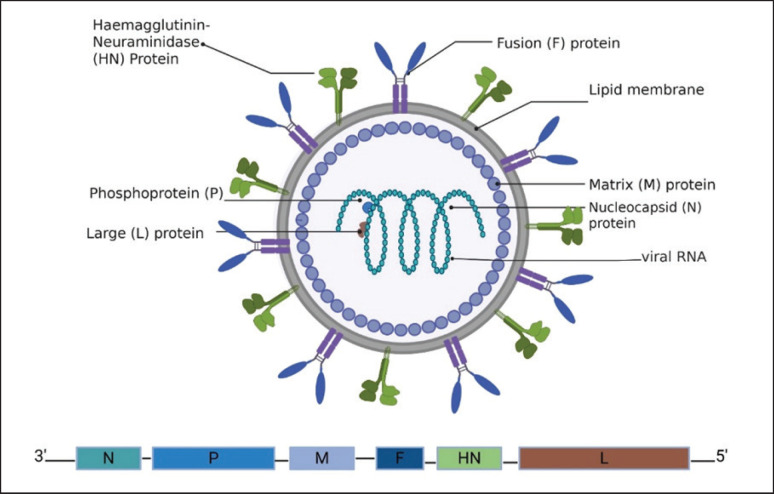
Unintended consequences of virus eradication.

Disease eradication represents an ambitious objective that requires genuine, coordinated efforts across multiple sectors. However, achieving eradication is not the end; the post-eradication era presents challenges that are equally, if not more, complex than those encountered during the eradication phase. These challenges were identified following the successful eradication of the smallpox virus in humans and rinderpest in cattle, and risk assessments were conducted years after the eradication process. The possible unintended consequences of virus eradication are summarized in Figure 2.

### Virus reemergence after vaccination

For example, monkeypox virus re-emergence and severe consequences are thought to occur in part because many populations were immunologically naïve after the cessation of smallpox vaccination in the final step of the eradication process [82]. The risk factor highlighted here is that some vaccines provide an umbrella of protection from the intended virus and some of its relatives. Therefore, the cessation of vaccination should be preceded by a careful assessment of the possible viruses that are masked and may emerge or reemerge. Some reports suggest the use of smallpox vaccines of the third generation in lab workers dealing with monkeypox virus [11,83].

In the case of the PPR virus, some research has focused on the virus’s ability to infect hosts from unusual species [11], which may occur in response to the cessation of the vaccination process. This host expansion can offer valuable lessons for consideration in current vaccination programs.

Some debate has arisen regarding the cessation of vaccination for the progressive control of poliovirus following reports of wild poliovirus and the rare reemergence of circulating vaccine-derived poliovirus [78,84-86]. Another report suggested that FMD-free status without vaccination should be equivalent to free status with vaccination in terms of sanitary protection [87,88]. In the case of the PPR virus, there is still no DIVA vaccine available for widespread application, especially in endemic settings. Therefore, should WOAH consider accepting “free with vaccination” as a final step in PPR eradication campaigns on some occasions?

Fear of viral resurgence or reintroduction after eradication, as seen with poliovirus, remains a significant concern [87]. Rinderpest remains a threat despite its eradication. The WOAH/FAO has made efforts to designate Rinderpest Holding Facilities and encouraged them to either sequence and then destroy them or keep them under control. Nevertheless, at least 7 laboratories still hold RPVs in their facilities without being controlled or inspected, which threatens the overall eradication process [17]. Owing to the close relationship between the rinderpest virus and the PPR virus, their control is an interconnected process.

### Predictive modeling role in foreseeing the possible outcomes of PPR eradication

Identifying and targeting high-risk populations through vaccination campaigns informed by the estimation of context-specific PPRV transmission levels would not only reduce the cost of PPR eradication but also, by setting more achievable vaccination coverage, increase the likelihood of success in eradication and post-eradication. To achieve this purpose, several studies have focused on modeling PPR transmission. In Ethiopia, a mathematical model was developed to study the transmission of PPR across Ethiopian villages. The goal of this study was to determine whether pastoral or highland areas are more prevalent in PPR. It was found that pastoral regions maintain PPR, and moving animals for trade from these regions carries a high risk of virus transmission. This study has both local and global effects; the trade of sheep and goats from the Ethiopian lowlands into neighboring countries and Gulf states occurs frequently [80]. The study by Gao et al. [89] carries significant importance, as it is the first to use modeling to demonstrate the possibility of cross-border transmission of PPR between wild and domestic animals. In the future, it will play a crucial role in monitoring the PPR epidemic and preventing its cross-border transmission [89].

Table 3 summarizes other models used for PPR in the literature and their potential applications in the eradication/post-eradication era.

**Table 3.** Another used models for PPR in the literature and their potential use to help the eradication/ post eradication era.

The model name	Use	Method	Country	Impact on eradication/ post eradication era	References
North American Animal Disease Spread Model (NAADSM).	Predict spread of PPR to RK ‘free zone’ from endemic zone.	Input data: location and population of SR farms. It uses: employs the stochastic simulations of the between-farm disease spread predicated on the SIR compartmental epidemic model.	Republic of Kazakhstan	Study the impact of diferent control and prevention measures on the spread of PPR as well as to assess the potential economic damage.	[93]
A deterministic mathematical model.	Investigate the impact of imperfect PPR vaccines and restocked small ruminants on the transmission dynamics of PPR	Incorporating vaccinated and restocked subpopulations into the classical SEIR model	Ethiopia	Emphasize that appropriate vaccination alone is insufficient to control and eradicate PPR in the region. Implementing strict movement restrictions and biosecurity measures are necessary.	[94]
Incorporating vaccinated and restocked subpopulations classical SEIR model	Transmission dynamics of PPR	Incorporating vaccinated and restocked subpopulations	Nigeria	The source of infection should be immediately removed either it was an inected aniamal quarantine or disinfection of in-contact premsises.	[95]
herd-level, event-driven model of PPR, using memoryless state transitions,	to study how the virus propagates through a herd, simulate the effectiveness of various control strategies	uses a set of matrixes describing discrete-state variables to define the characteristics of the animals in the herd and the condition of the herd with respect to PPR disease states.	Afghanistan	Reducing the amount of time from the identification of PPR in a herd to the vaccination of the herd will radically reduce the number of deaths that result from PPR.	[96]

Ecological Niche Modelling is a computational, data-driven method broadly applied in ecology, evolutionary studies, and conservation biology. It employs algorithms to evaluate how closely environmental conditions in a given geographic area resemble those at sites where a species or phenomenon has been observed. The approach is most commonly used to forecast species distributions by combining occurrence records (georeferenced detection points) with environmental variables provided through Geographic Information Systems (GIS) layers [94]. We suggest that such a model can be suggested to policymakers in areas where vaccination is being stopped to give insights into the potential risk of PPR transmission and eruptions in these regions, to focus on animal control, rapid testing, and slaughter of PPR reactors with proper disinfection and quarantine.

Should prediction mathematics give insights into the possible outcomes that can occur after the eradication of PPR, based on our previous experience with rinderpest, poliovirus, and smallpox viruses that were successfully eradicated? This was used for predicting PPR in various wildlife-domestic ruminant interfaces, both locally and worldwide [90-92,95].

Modeling tools should be tailored and used to translate our concerns of new emergence into clearly defined risk points that can guide the development of targeted actions and strategic planning. An Ecological Niche Modelling study implemented in 2020 has predicted PPR incursion in Italy [96]. It is known that PPR has emerged in Europe in 2024–2025; although no reports have mentioned that Italy has been affected, this indicates that this kind of predictive modeling can help proactively. We recommend using Ecological Niche Modelling to predict areas that may be at risk of PPR reemergence after eradication, taking into account climate change, animal susceptibility, distribution, and other relevant factors.

Transmission models are used for various vaccination strategies, in addition to Epimodel, which can be employed to model transmission dynamics and spillover possibilities [17,97,98]. All these research efforts should be communicated properly to stakeholders and policymakers.

Application of modeling to predict unintended consequences of eradication: the resurgence of PPR in Europe during 2024–2025. It is demonstrated that ecological niche modeling exhibits superior performance in predicting PPR transmission and can be utilized to detect the potential for PPR transmission after eradication, as seen in Europe, where achieving PPR-free status is anticipated in the near future.

## Conclusion

PPR is a virus of concern. In the remaining 5 years, until the announcement of complete eradication, some steps still needed to be taken. The virological scope includes the mass production of DIVA vaccines and the provision of sufficient doses to endemic settings. DIVA vaccines should be supported with DIVA ELISA kits to achieve correct serosurveillance. These kits should be validated on all possible hosts of PPR. Wrong vaccine practices include skipping nomadic animals and herd reconstruction without labelling for each animal that received the vaccine. Testing of all possible hosts by deep sequencing for the detection of PPR presence and its shedding.

## Recommendations

· Accelerate the distribution and validation of the DIVA vaccine among species.

· Introduce predictive modeling into national initiatives to eradicate PPR.

· Consider the approach of ‘’vaccination + surveillance’’ as a valid endgame for eradication and therefore accept ‘’free with vaccination’’ status as accepted in the eradication last phase.

## List of abbreviations

AGP, anti-genome promoter region; CDV, canine distemper virus; CPE, Cytopathic effects; CIRAD, Centre de coopération internationale en recherche agronomique pour le développement; DMSO, Dimethyl sulfoxide; ELISA, Enzyme linked Immunosorbent Assay; FAO, Food and Agriculture Organization; FMD, foot-and-mouth disease; GF-TADS, Global Framework for the Progressive Control of Transboundary Animal Diseases; GEP, global eradication program; FeMV, feline morbillivirus; PCR, polymerase chain reaction; PAMPs, pathogen-associated molecular patterns; PMAT, PPR monitoring and assessment tool; PPR, peste des petits ruminants; PPRV, peste des petits ruminants virus; RPV, rinderpest virus; SLAM, signaling lymphocytic activation molecule; SRMV, small ruminant morbillivirus; ssRNA, single-stranded RNA; RT-PCR, real-time polymerase chain reaction.
